# Rest

**Published:** 1869-07

**Authors:** 


### REST.

WHAT TO DO THIS SUMMER.

Lay off during hot weather. Let your business suffer rather than health. The laboring man and mechanic have sufficient exercise to keep their bodies in a healthful condition. But the professional man and the merchant, whose lâbor is principally "head-work," require rest of brain, or the body soon becomes diseased. The business man who has been laboring early and late during the winter just past, in order to support the extravagance of a fashionable, and therefore unhealthy family, experiences a sense of lassitude, a tired, "worn out" feeling, as the warm weather approaches. He thinks he would like to rest from his business awhile, but cannot, as long as he is present with it, and is fearful to leave it, lest it suffer in his absence. And so he tugs along through the hot summer months, year after year, until he becomes an irritable, peevish, withered-up, and prematurely *old* man. He then seeks relief from the hands of his physician, who tells him that he has "softening of the brain," from too much mental fatigue, and that he should have taken rest from his business at least one month every summer, in order to keep his brain and nervous system in good working order. In this state he lingers for two or three years longer, and finally dies, from consumption, paralysis, or "premature decay," when, had he taken the necessary resting spells once every year, he *ought* to have been just in the prime of life.

We know how difficult it is to attract some business men from their desks by offering them pleasure excursions. *Their* only pleasure is in business, and they are unable to see any sort of sport in anything else.

Tell them of trout-fishing, the beautiful scenery, of certain mountains, and the splendid appetite one secures by a week's sojourn in the mountain wilds, and they will only laugh at your enthusiasm. Tell them of the splendid blue-fishing, invigorating sea-air, and glorious surf-bathing, of some quiet little village on the coast, and how much better you feel for a month's rollicking there, and these grim, withered-up old misers will grit their false teeth, scratch their rusty wigs, and tell you money is not made in that way—as though a man should live for nothing but *money* in this world. *Such* men require to be *ordered* from their business by their medical attendant, and be required to take rest in some such place, as you would compel a child to take a dose of nauseous medicine.

But, young men, those who are just forming your business habits, it is your duty to arrange your business affairs so as to have *some* recreation and rest every summer. Do not go to fashionable watering places, where you are compelled to wear nobby clothes, and your wife forced to dress within an inch of her life —where you "live high" and dance all night, but go to some *quiet* place—some farm-house, if possible, where you can go in your shirt-sleeves, and your wife and children dress in calico—where the daily tramps, fishing excursions and the bracing sea-air will cause your appetites to appreciate the plain fare set before you. Go in groups—two or three families going in company, and have a real jolly time of it. Such a trip, of a month's duration, may cost you one hundred dollars, but will save you a thousand in doctor's bills and funeral expenses.

